# Undifferentiated Embryonal Sarcoma of the Liver in Children Versus Adults: A National Cancer Database Analysis ^†^

**DOI:** 10.3390/cancers13122918

**Published:** 2021-06-11

**Authors:** Ioannis A. Ziogas, Irving J. Zamora, Harold N. Lovvorn III, Christina E. Bailey, Sophoclis P. Alexopoulos

**Affiliations:** 1 Department of Surgery, Division of Hepatobiliary Surgery and Liver Transplantation, Vanderbilt University Medical Center, Nashville, TN 37232, USA; ioannis.a.ziogas@vumc.org; 2 Department of Pediatric Surgery, Monroe Carell, Jr. Children’s Hospital at Vanderbilt, Nashville, TN 37232, USA; irving.zamora@vumc.org (I.J.Z.); harold.lovvorn@vumc.org (H.N.L.III); 3 Department of Surgery, Division of Surgical Oncology, Vanderbilt University Medical Center, Nashville, TN 37232, USA; christina.e.bailey@vumc.org

**Keywords:** embryonal sarcoma, liver sarcoma, hepatic sarcoma, liver cancer, National Cancer Database

## Abstract

**Simple Summary:**

Undifferentiated embryonal sarcoma of the liver (UESL) is the third most common type of liver malignancy in the pediatric population, following hepatoblastoma and hepatocellular carcinoma. In comparison to children, UESL is an extremely rare malignancy in adults. Although historically treatment was limited to surgical resection and survival was poor, the combination of surgical treatment and chemotherapy recently has led to improved survival. We attempted to examine the characteristics and outcomes of children and adults with UESL in a contemporary U.S. cohort. We showed that children demonstrate favorable survival with multimodal treatment, while adults demonstrate inferior outcomes and future research endeavors should focus on refining currently available treatment modalities for adults with UESL.

**Abstract:**

This study evaluates the clinicopathological characteristics and outcomes of children vs. adults with undifferentiated embryonal sarcoma of the liver (UESL). A retrospective analysis of 82 children (<18 years) and 41 adults (≥18 years) with UESL registered in the National Cancer Database between 2004–2015 was conducted. No between-group differences were observed regarding tumor size, metastasis, surgical treatment, margin status, and radiation. Children received chemotherapy more often than adults (92.7% vs. 65.9%; *p* < 0.001). Children demonstrated superior overall survival vs. adults (log-rank, *p* < 0.001) with 5-year rates of 84.4% vs. 48.2%, respectively. In multivariable Cox regression for all patients, adults demonstrated an increased risk of mortality compared to children (*p* < 0.001), while metastasis was associated with an increased (*p* = 0.02) and surgical treatment with a decreased (*p* = 0.001) risk of mortality. In multivariable Cox regression for surgically-treated patients, adulthood (*p* = 0.004) and margin-positive resection (*p* = 0.03) were independently associated with an increased risk of mortality. Multimodal treatment including complete surgical resection and chemotherapy results in long-term survival in most children with UESL. However, adults with UESL have poorer long-term survival that may reflect differences in disease biology and an opportunity to further refine currently available treatment schemas.

## 1. Introduction

Undifferentiated embryonal sarcoma of the liver (UESL) is the third most common type of liver malignancy in the pediatric population, following hepatoblastoma and hepatocellular carcinoma, and became a distinct pathology after the report of Stocker and Ishak in 1978 [1]. Due to a low estimated annual incidence of one per million [2], there are only a few reported cases in the literature. It is a highly aggressive malignancy of mesenchymal origin with a tendency for local and distant metastasis [3]. Typically, UESL are large tumors with an average diameter of 10–30 cm [4]. The clinical manifestations (abdominal pain, fever, anorexia) and the radiographic characteristics (solid and cystic components) are nonspecific, and thus UESL may pose a significant diagnostic challenge that can lead to a delay in appropriate management [3,4,5,6,7,8,9]. Although historically treatment was limited to surgical resection and overall survival (OS) was poor [1], the combination of surgical treatment and chemotherapy recently has led to improved OS [10,11,12,13,14,15].

In comparison to children, UESL is an extremely rare malignancy in adults [16]. According to a recent systematic review and pooled analysis, less than 90 adult UESL cases have been published between 1973–2019 [6]. The authors reported that, compared to children, a higher proportion of adults presented with metastatic disease and received no surgical treatment with 5-year OS rates of 49.5% vs. 79.9%, respectively [6]. However, that cohort was comprised of patients managed over different time periods and in different healthcare systems. Therefore, we aimed to evaluate the characteristics and compare OS between pediatric and adult UESL patients in the United States over a contemporary era, and to identify risk factors of mortality. We hypothesized that adults with UESL present with more advanced disease and demonstrate inferior OS compared to children with UESL.

## 2. Materials and Methods

### 2.1. Data Source and Patient Population

We included all patients with UESL registered in the National Cancer Database (NCDB) between 2004–2015. The NCDB is a joint project of the Commission on Cancer of the American College of Surgeons and the American Cancer Society and incorporates about 70% of all newly diagnosed cancers in more than 1500 hospitals accredited by the Commission on Cancer in the United States [17]. It includes data on demographics, clinicopathological characteristics, tumor characteristics, management, and survival [18].

**Figure 1:**
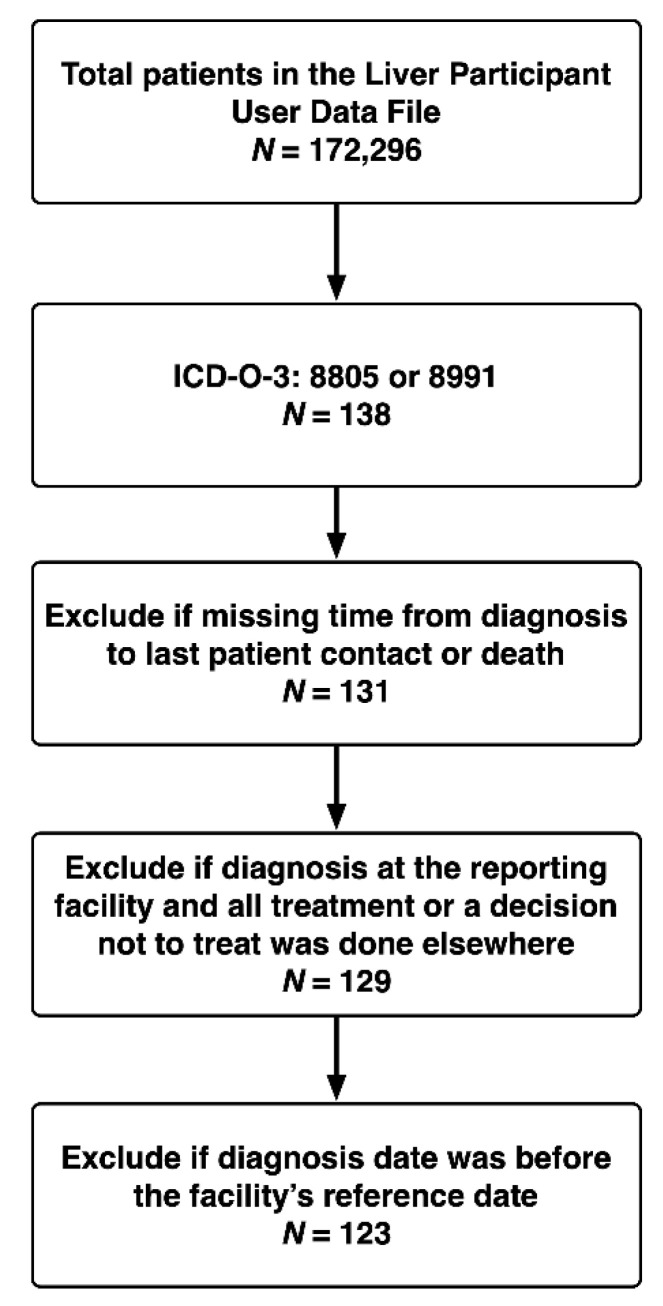
Assembly of Analysis Cohort.

For the present study, we used the NCDB Participant Use Data File to identify both children (<18 years) and adults (≥18 years) with UESL using the International Classification of Diseases for Oncology, 3rd Edition, with the combination of liver site code “C22.0” and the histology codes “8805” and “8991”. We excluded patients with missing data about the time between diagnosis and death or last patient contact, or with missing data about the vital status at last patient contact. Figure 1 depicts our cohort assembly. Institutional Review Board approval was not required as all data were de-identified.

### 2.2. Covariates and Outcomes

We extracted the following patient demographic data: age, sex, race, insurance status, and year of diagnosis. Clinicopathological and treatment-related data that were extracted included tumor size, regional lymph node status, metastasis, extent of surgical treatment (wedge/segmental resection, lobectomy, extended lobectomy, resection not otherwise specified, liver transplantation (LT)), resection margin status, receipt of chemotherapy, systemic therapy (chemotherapy)-surgery sequence, and receipt of radiotherapy. We used the number of days between diagnosis and chemotherapy initiation when the systemic therapy (chemotherapy)-surgery sequence data were not available. OS was defined as the time from diagnosis until death or last patient contact and was our primary outcome.

### 2.3. Statistical Analysis

Continuous variables were summarized as medians (interquartile ranges (IQRs)) and were compared using the Mann-Whitney U test, while categorical variables were summarized as frequencies (%) and were compared using the chi-square test. The Kaplan-Meier method was employed for survival analysis, and we performed pairwise comparisons using the log-rank test. We also fitted Cox regression models to obtain the hazard ratio (HR) and a 95% confidence interval (CI). In order to avoid the inferential limitations of selecting variables for multivariable models based on stepwise procedures or univariable comparisons, we prespecified the variables to be included in our multivariable models [19]. Our first multivariable model assessing factors associated with mortality in the entire cohort incorporated the following variables: age group (children vs. adults), metastatic status at the time of diagnosis, surgical treatment, chemotherapy, and radiation. Our second multivariable model assessing factors associated with mortality in the surgical cohort incorporated the following variables: age group (children vs. adults), metastatic status at the time of diagnosis, chemotherapy, radiation, tumor size, and surgical margin status. All statistical analyses were conducted using Stata IC 16.0 (StataCorp LLC, College Station, TX, USA). All *p*-values were based on two-sided statistical tests, and significance was set at <0.05.

## 3. Results

### 3.1. Patient Demographics and Treatment Modalities

Overall, 123 patients with UESL (82 children and 41 adults) were identified in the NCDB (Table 1). The median age was 11.0 years (IQR: 6.0–23.0), more than half of the patients were female (57.7%), and 69.9% were of Caucasian race. Median tumor size was 14.0 cm (IQR: 10.0–16.0), metastasis at diagnosis was seen in 13.8% of the patients, and 82.9% of them underwent surgical treatment (LT in 4 children). When the cohort was separated into age groups, children were less often female than adults (50.0% vs. 73.2%; *p* = 0.01), while no statistically significant differences were observed between the two comparison groups regarding race, insurance status, tumor size, regional lymph node status, metastasis, surgical treatment and margin status, and receipt of radiation. However, the proportion of children who received chemotherapy was higher than that of the adults (92.7% vs. 65.9%; *p* < 0.001). Of the 64 children receiving both surgery and chemotherapy, 14 (22%) had neoadjuvant, 37 (58%) had adjuvant, and 13 (20%) had both neoadjuvant and adjuvant therapy. Of the 23 adults receiving both surgery and chemotherapy, 1 (4%) had neoadjuvant and 22 (96%) had adjuvant therapy.

### 3.2. Overall Survival

**Figure 2:**
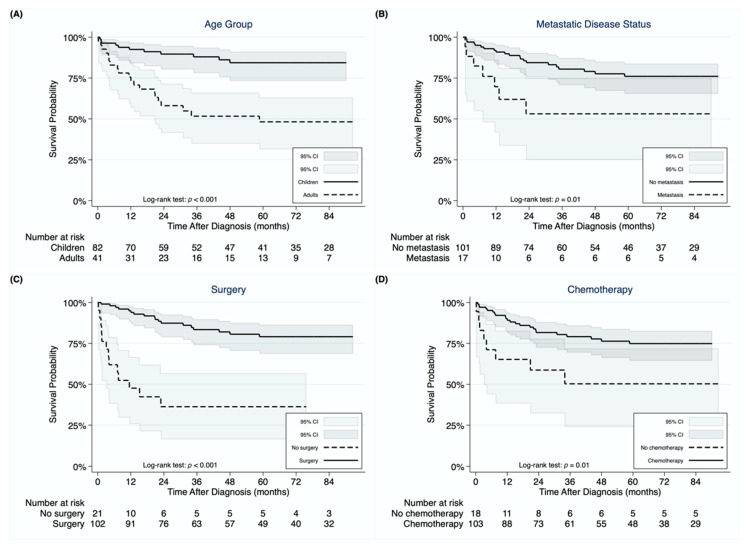
Kaplan-Meier curves demonstrating differences in overall survival for undifferentiated embryonal sarcoma of the liver between children and adults (**A**), between patients with and without metastasis at diagnosis (**B**), between patients undergoing surgery or not (**C**), and between patients receiving chemotherapy or not (**D**).

The 1-, 3-, and 5-year OS rates in the entire cohort were 86.8%, 75.4%, and 71.7%, respectively. Children demonstrated superior OS compared to adults (log-rank test: *p* < 0.001) with 5-year OS rates of 84.4% and 48.2%, respectively (Figure 2A). Metastatic disease resulted in decreased survival compared to non-metastatic disease (5-year OS rates of 53.1% and 76.0%, respectively; log-rank test: *p* = 0.01; Figure 2B). Surgically treated patients exhibited superior OS compared to those treated non-operatively (5-year OS rates of 79.1% and 36.3%, respectively; log-rank test: *p* < 0.001; Figure 2C). Patients receiving chemotherapy exhibited superior OS compared to those who did not receive chemotherapy (5-year OS rates of 74.9% and 50.3%, respectively; log-rank test: *p* = 0.01; Figure 2D). Four children who underwent neoadjuvant chemotherapy and LT were alive over a follow-up period of 17–80 months post-LT.

In multivariable Cox regression analysis for the entire cohort (Table 2), adults demonstrated 5.35 times higher risk of mortality (95% CI: 2.24–12.77; *p* < 0.001) compared to children, while metastasis was also associated with an increased risk of mortality (HR = 3.34, 95% CI: 1.20–9.29; *p* = 0.02). On the other hand, surgical treatment was associated with a survival benefit (HR = 0.21, 95% CI: 0.09–0.52; *p* = 0.001). We next performed multivariable Cox regression analysis to identify risk factors of mortality in surgically-treated patients (Table 3). Being adult (HR = 10.68, 95% CI: 2.10–54.33; *p* = 0.004) and having a margin-positive resection (HR = 5.41, 95% CI: 1.18–24.75; *p* = 0.03) were identified as parameters independently associated with an increased risk of mortality.

## 4. Discussion

UESL accounts for around 9–15% of pediatric liver cancers [1,4] and is only rarely reported in adults [16,20]. The most common treatment consists of surgical resection with the recent addition of chemotherapy [2,6]. The present study is unique in its inclusion of the largest number of UESL patients diagnosed and treated in a contemporary era and is the first to compare OS between children and adults with UESL in the United States. Moreover, our findings demonstrated that although the majority of patients are offered surgical treatment, adults with UESL were less commonly treated with chemotherapy compared to children despite similar tumor size and rates of metastatic disease. When adjusting for covariates, adults had a five- and ten-fold increased risk of mortality compared to children in the entire and surgical cohorts, respectively. Additionally, margin-negative resection is essential to achieve long-term OS.

Prognosis for UESL was historically considered to be dismal [1,21,22,23]. More recent reports have shown an improvement in OS [10,11,13,15,24,25,26,27], which has been mostly attributed to the increasing use of chemotherapy in conjunction with surgical treatment [2,10,11]. In a recent systematic review and pooled analysis of 308 patients (219 children and 89 adults), the 5-year OS rate was 65.8%, and more specifically 70.4% for those receiving surgical treatment vs. 6.6% for those receiving no surgical treatment [6]. These authors further identified margin-negative resection, receipt of chemotherapy, and childhood as factors independently associated with an improved survival in surgically-treated UESL patients. Using more homogeneous and contemporary data from the U.S., we showed that the 5-year OS rate for all UESL patients in the United States was 71.7%, and 79.1% for those treated surgically vs. 36.3% for those not treated surgically. Although we also showed the association of margin-negative resection and childhood with improved OS, our results regarding the importance of adding chemotherapy and/or radiotherapy to the surgical treatment plan, as well as the setting of administration, were inconclusive. A recent literature review showed that approximately 26% of UESL patients receive neoadjuvant chemotherapy, while more than 70% receive adjuvant chemotherapy [27], which is consistent with our findings. A recent multicenter study from Europe reported on 25 patients who underwent primary resection (*n* = 12) or neoadjuvant chemotherapy (*n* = 13; delayed resection was employed in 8, while the other 5 remained inoperable); 20 patients received adjuvant chemotherapy [28]. The authors concluded that complete resection is vital for the management of UESL and that surgery plus multiagent chemotherapy can yield favorable long-term outcomes [28]. Currently, no chemotherapy protocol is established for UESL [28], while the most commonly used agents include vincristine, ifosfamide, and doxorubicin [6,27,28]. Additionally, radiation therapy has only been scarcely used for UESL according to both our findings (17%) and the literature (15–16%) [2,6,27]. Due to the lack of recent studies and the low incidence of this disease, multicenter prospective studies comparing surgery alone vs. surgery plus chemotherapy over a contemporary era are required to draw more robust conclusions about the effect of chemotherapy on OS. Additionally, immunotherapy constitutes another area that future research endeavors could focus on [29].

In general, UESL patients present with an abdominal mass and pain along with anorexia and weight loss [6,30]. Laboratory liver tests and tumor markers are typically normal, while elevated liver enzymes and cancer antigen 125 have been described in a few cases [3,4,30,31]. Additionally, fever and an increase in C-reactive protein, erythrocyte sedimentation rate, and leukocytes may be seen in cases of hemorrhage or necrosis in the tumor [4]. Macroscopically, UESLs are well-circumscribed with cystic and solid components of gray-white gelatinous areas with or without red and yellow hemorrhagic and necrotic parts [30,32]. Microscopically, spindle or stellate cells with ill-defined borders, hyperchromatic nuclei, inconspicuous nucleoli, and eosinophilic PAS-positive and diastase-resistant cytoplasmic globules along with multinucleated giant cells in a myxoid matrix are typically seen [3,30,32]. Although the immunohistochemical pattern is not specific, markers of histiocytic, muscle, and epithelial origin may be identified, such as vimentin, alpha-1 antitrypsin, desmin, and CD68 [4,33,34,35,36].

Although the importance of resection with negative margins in OS is unquestionable [28], data suggest that patients with unresectable UESL can demonstrate long-term OS with LT. In our analysis, four children undergoing neoadjuvant chemotherapy plus LT were alive at 17–80 months post-LT. A United Network of Organ Sharing database analysis showed that out of 12 children undergoing LT for UESL, 11 survived and 1 died postoperatively [27]. In an older NCDB analysis, all 10 children undergoing LT were alive at 5 years [2]. A systematic review also showed that out of 14 patients (10 children and 4 adults) undergoing LT for UESL, 3 died [6]. Several other case reports and case series have also demonstrated the ability to achieve long-term survival for UESL with chemotherapy and either primary or salvage LT [12,14,26,37,38]. These findings validate the role of LT in the management of unresectable UESL.

Our study is the first to investigate the clinicopathological characteristics and outcomes of both children and adults with UESL in the United States over a recent era. We showed that children with UESL survive longer than adults with the 5-year OS rates being 84.4% and 48.2%, respectively. This survival benefit persisted even when adjusting for covariates in both the entire cohort and in surgically-treated patients. The only previous study to investigate this comparison was a heterogenous cohort of systematically reviewed patients (1973–2019), which reported a 5-year OS of 79.9% in children and 49.5% in adults with UESL [6]. The authors also reported that this benefit persisted in the multivariable analysis of patients undergoing partial hepatectomy [6]. Based on these findings, although similar in histology, UESL appears to be not only more rare but also more aggressive in adults compared to children irrespective of metastatic disease status and receipt of surgery and/or chemotherapy. Further research needs to be pursued to unveil the underlying etiology for this disparity in disease biology and to refine currently available treatment schemas for adults with UESL. It remains apparent that UESL should be considered in the differential diagnosis in children and particularly adults with an atypical presentation of a large liver lesion to schedule surgical treatment in a timely fashion.

The present study has certain limitations, mostly inherent to the nature of retrospective database analyses. The NCDB does not capture some clinically important data fields, such as staging of liver tumors according to the Children’s Oncology Group or pretreatment extent of disease, chemotherapy regimen, alpha-fetoprotein level, and response of the tumor to chemotherapy. Therefore, we were not able to assess the impact of these variables on OS, while we also could not evaluate recurrence-free survival as tumor recurrence data were not available in the NCDB. Additionally, due to the rarity of the disease, the study sample is relatively small, and caution is warranted in the interpretation of the results.

## 5. Conclusions

In conclusion, UESL constitutes a rare entity typically exceeding 10 cm in tumor diameter. Although mostly seen in children, it should always be considered in the differential diagnosis of an atypical liver lesion in adults, due to its highly aggressive behavior and its association with an increased risk of mortality in this age group. Prompt management with surgical resection in cases of resectable UESL or LT in cases of unresectable or locally recurrent UESL is of paramount importance to achieve long-term OS. Multimodal treatment results in long-term OS in most children with UESL. However, adults with UESL have poorer outcomes that may reflect differences in disease biology and an opportunity to further refine currently available treatment modalities in this age group.

## Figures and Tables

**Table 1:** Cohort characteristics.

Characteristics	Total (*n* = 123)	Children (*n* = 82)	Adults (*n* = 41)	*p* Value
Age (years)	11.0 (6.0–23.0)	7.5 (5.0–11.0)	36.0 (23.0–62.0)	<0.001
Sex				0.01
Female	71 (57.7%)	41 (50.0%)	30 (73.2%)	
Male	52 (42.3%)	41 (50.0%)	11 (26.8%)	
Race				0.22
African American	24 (19.5%)	13 (15.9%)	11 (26.8%)	
Caucasian	86 (69.9%)	60 (73.2%)	26 (63.4%)	
Other	9 (7.3%)	5 (6.1%)	4 (9.8%)	
Unknown	4 (3.3%)	4 (4.9%)	0 (0.0%)	
Insurance status				0.97
Not insured	3 (2.4%)	2 (2.4%)	1 (2.4%)	
Private	63 (51.2%)	41 (50.0%)	22 (53.7%)	
Public	53 (43.1%)	36 (43.9%)	17 (41.5%)	
Unknown	4 (3.3%)	3 (3.7%)	1 (2.4%)	
Tumor size (cm) (*n* = 108)	14.0 (10.0–16.0)	14.0 (11.0–15.9)	14.8 (7.6–19.5)	0.90
Regional lymph node status				0.44
Negative	30 (24.4%)	18 (22.0%)	12 (29.3%)	
Positive	4 (3.3%)	3 (3.7%)	1 (2.4%)	
No nodes were examined	86 (69.9%)	60 (73.2%)	26 (63.4%)	
Unknown	3 (2.4%)	1 (1.2%)	2 (4.9%)	
Metastasis at diagnosis				0.33
No	101 (82.1%)	65 (79.3%)	36 (87.8%)	
Yes	17 (13.8%)	14 (17.1%)	3 (7.3%)	
Unknown	5 (4.1%)	3 (3.7%)	2 (4.9%)	
Chemotherapy				<0.001
No	18 (14.6%)	4 (4.9%)	14 (34.2%)	
Yes	103 (83.7%)	76 (92.7%)	27 (65.9%)	
Unknown	2 (1.6%)	2 (2.4%)	0 (0.0%)	
Radiation				0.20
No	101 (82.1%)	64 (78.1%)	37 (90.2%)	
Yes	21 (17.1%)	17 (20.7%)	4 (9.8%)	
Unknown	1 (0.8%)	1 (1.2%)	0 (0.0%)	
Surgical treatment				0.31
No	21 (17.1%)	12 (14.6%)	9 (22.0%)	
Yes	102 (82.9%)	70 (85.4%)	32 (78.0%)	
Type of surgical treatment (*n* = 102)				0.29
Wedge/segmental resection	30 (29.4%)	22 (31.4%)	8 (25.0%)	
Lobectomy	43 (42.2%)	28 (40.0%)	15 (46.9%)	
Extended lobectomy	14 (13.7%)	7 (10.0%)	7 (21.9%)	
Resection, not otherwise specified	11 (10.8%)	9 (12.9%)	2 (6.3%)	
Liver transplantation	4 (3.9%)	4 (5.7%)	0 (0.0%)	
Margin status (*n* = 102)				1.00
No residual tumor	71 (69.6%)	48 (68.6%)	23 (71.9%)	
Residual tumor	17 (16.7%)	12 (17.1%)	5 (15.6%)	
Not evaluable/unknown	14 (13.7%)	10 (14.3%)	4 (12.5%)	
Chemotherapy/surgery sequence (*n* = 102)				<0.001
None	13 (12.8%)	4 (5.7%)	9 (28.1%)	
Neoadjuvant	15 (14.7%)	14 (20.0%)	1 (3.1%)	
Adjuvant	59 (57.8%)	37 (52.9%)	22 (68.8%)	
Neoadjuvant and adjuvant	13 (12.8%)	13 (18.6%)	0 (0.0%)	
Unknown	2 (2.0%)	2 (2.9%)	0 (0.0%)	
Follow-up time after diagnosis (months)	48.1 (20.2–92.6)	61.2 (22.9–102.9)	29.6 (12.0–71.4)	0.007

**Table 2:** Risk factors associated with mortality in the entire cohort.

Characteristics	*n*	Univariable		*n*	Multivariable (*n* = 115)	
		HR (95% CI)	*p* Value		HR (95% CI)	*p* Value
Age group						
Children	82	Reference	-	76	Reference	-
Adults	41	4.36 (2.08–9.11)	<0.001	39	5.35 (2.24–12.77)	<0.001
Surgical treatment						
No	21	Reference	-	18	Reference	-
Yes	102	0.16 (0.08–0.34)	<0.001	97	0.21 (0.09–0.52)	0.001
Chemotherapy						
No	18	Reference	-	16	Reference	-
Yes	103	0.36 (0.16–0.81)	0.01	99	0.59 (0.22–1.60)	0.30
Radiation						
No	101	Reference	-	94	Reference	-
Yes	21	0.72 (0.25–2.07)	0.55	21	1.18 (0.39–3.56)	0.77
Metastasis at diagnosis						
No	101	Reference	-	98	Reference	-
Yes	17	2.82 (1.19–6.66)	0.02	17	3.34 (1.20–9.29)	0.02

Abbreviations: CI = confidence interval; HR = hazard ratio.

**Table 3:** Risk factors associated with mortality in surgically-treated patients.

Characteristics	*n*	Univariable		*n*	Multivariable (*n* = 79)	
		HR (95% CI)	*p* Value		HR (95% CI)	*p* Value
Age group						
Children	70	Reference	-	53	Reference	-
Adults	32	4.95 (1.85–13.23)	0.001	26	10.68 (2.10–54.33)	0.004
Surgical margins						
No residual tumor	72	Reference	-	64	Reference	-
Residual tumor	17	2.08 (0.71–6.08)	0.18	15	5.41 (1.18–24.75)	0.03
Tumor size (cm)	92	1.02 (0.99–1.04)	0.09	79	1.00 (0.97–1.04)	0.89
Chemotherapy						
Neoadjuvant and adjuvant	13	Reference	-	12	Reference	-
None	13	1.13 (0.23–5.62)	0.88	9	0.43 (0.04–5.26)	0.51
Neoadjuvant	15	0.55 (0.09–3.28)	0.51	9	1.24 (0.11–14.50)	0.86
Adjuvant	59	0.73 (0.20–2.65)	0.63	49	0.25 (0.03–1.84)	0.18
Radiation						
No	82	Reference	-	63	Reference	-
Yes	19	0.92 (0.27–3.18)	0.90	16	0.51 (0.07–3.52)	0.49
Metastasis at diagnosis						
No	90	Reference	-	72	Reference	-
Yes	10	1.45 (0.33–6.31)	0.62	7	4.29 (0.59–30.89)	0.15

Abbreviations: CI = confidence interval; HR = hazard ratio.

## Data Availability

The data that support the findings of this study are available from the Commission on Cancer’s National Cancer Database (NCDB). They are de-identified patient level data that do not identify hospitals, health care providers, or patients as agreed to in the Business Associate Agreement that each Commission on Cancer accredited program has signed with the American College of Surgeons. Restrictions apply to the availability of these data, which were used under license for this study. Data are available at https://www.facs.org/quality-programs/cancer/ncdb/puf (accessed date: 21 January 2020) with the permission of the American College of Surgeons.
